# The laparoscopic management of simple hepatic cysts

**Published:** 2015

**Authors:** CA Stănescu, DN Păduraru, C Cirimbei, E Brătucu

**Affiliations:** * “Hopital Cantonal Fribourg”, Fribourg, Switzerland; ** ”Carol Davila” University of Medicine and Pharmacy, Bucharest, Romania; *** Surgery Clinic III, Emergency University Hospital, Bucharest, Romania; **** ”Alexandru Trestioreanu” Oncologic Institute, Bucharest, Romania

**Keywords:** laparoscopic management, simple hepatic cysts, hepatic polycystic disease

## Abstract

The hepatic polycystic disease represents a hereditary condition with a reduced prevalence in the general population, sometimes associated with polycystic kidney disease. We present a retrospective observational study applied to 49 patients. The study aimed to observe the laparoscopic surgery of simple hepatic cysts. Laparoscopic approach is a simple and successful surgery management of these types of cysts.

## Introduction

The liver fibrocystic conditions include the hepatic polycystic disease, congenital solitary liver cysts, Caroli disease and von Meyenburg complexes. The hepatic polycystic disease represents a hereditary condition [**1**]. Its prevalence in the general population is reduced, of approximately 3-5%. In most cases, the hepatic polycystic disease is diagnosed in adults, either in association with polycystic kidney disease, an autosomal dominant transmitted disease, or isolated as a hepatic polycystic disease. Its prevalence increases with age and the disease is more frequently found in the females [**2**].

From an etiopathogenic point of view, most simple hepatic cysts are congenital (primary), derived from an abnormal development of some isolated biliary ducts which lose their communication with the biliary main trunk.

## Material and method

Our study set the aim of analyzing the treatment applied to 49 patients by a retrospective observational study. The patients who fulfilled the inclusion criteria for the research study were admitted and treated in the Surgery Clinic of “Caritas” Clinical Hospital in Bucharest, between 1990 and 2008, and in “Saint Flour” Hospital in France, between 2000 and 2008.

All the 49 patients included in the clinical trial were selected according to clinical, biochemical and imagistic criteria for the hepatic cystic disease. The pre-operative imagistic diagnostic for all the patients was made by echography and computerized tomography or magnetic resonance imaging.

## Results

**Fig. 1:**
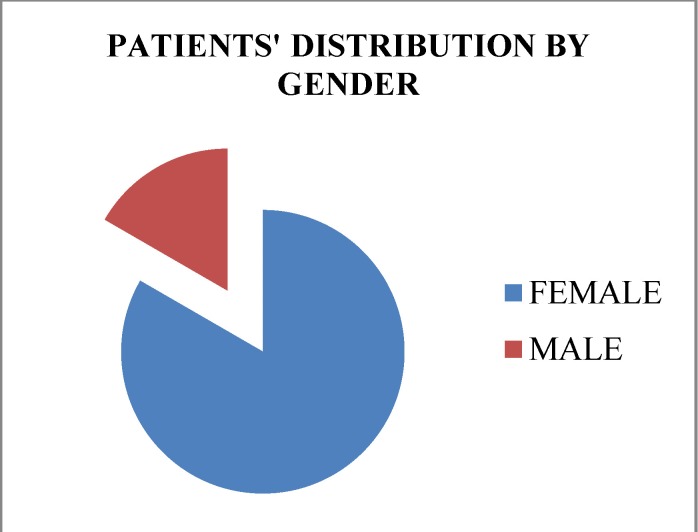
The patients’ distribution according to gender

Out of the 49 patients who fulfilled the inclusion criteria for the research study, 34 were females and 15 males (**Fig. 1**).

**Table 1:** The patients included in the study – age groups

Patients’ age (years)	General lot
Mean	52.5
Median	51
Standard deviation	14.499
Minim	24
Maxim	84

The patients included in the study were between 24 and 84 years old, with a mean age of 52.5 years old. (**Table 1**).

**Fig. 2:**
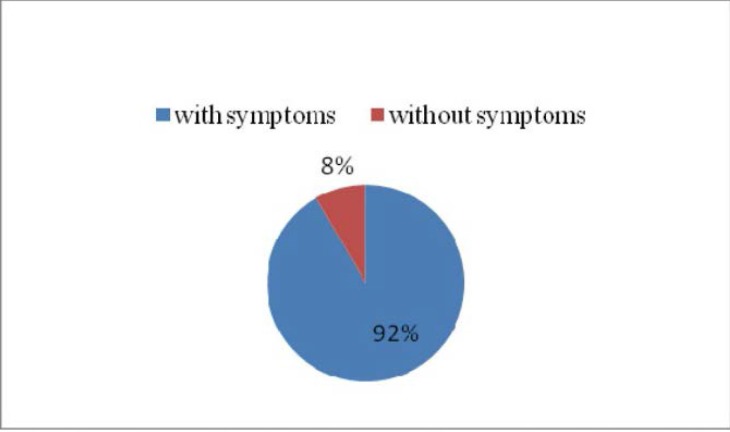
Clinical diagnosis – patients showing symptoms and patients without symptoms

Out of the 49 cases included in the study, 42 patients were asymptomatic (**Fig. 2**).

**Fig. 3:**
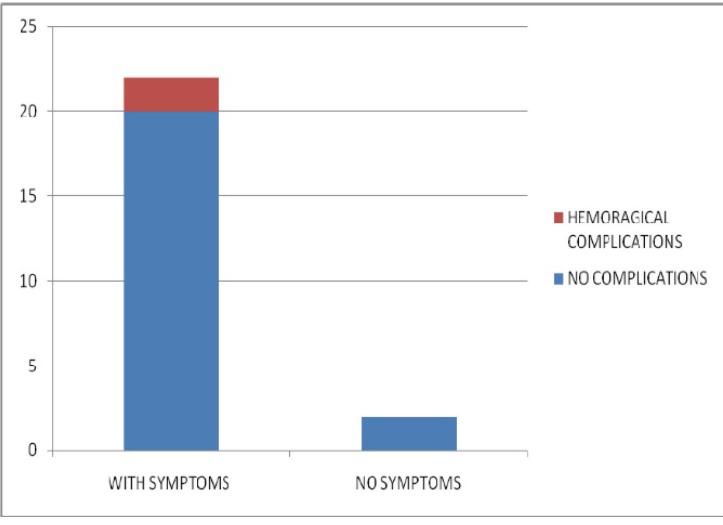
The patients’ distribution according to the presence or the absence of symptomatology

Out of 39 symptomatic patients, 4 presented for admission with hemorrhagic complications.

The biological changes were identified in only 4 patients, 20 other cases showing a normal biological preoperative status.

In all the cases included in the study, the imagistic diagnostic was in conjunction with computerized tomography or magnetic resonance imaging. The hepatic echography was considered the simplest diagnose method for solitary cysts and it was used in the research study. The solitary cysts were described as anechoic, ovoid or circular areas, well limited with minute edges and pronounced posterior echo.

**Fig. 4, 5:**
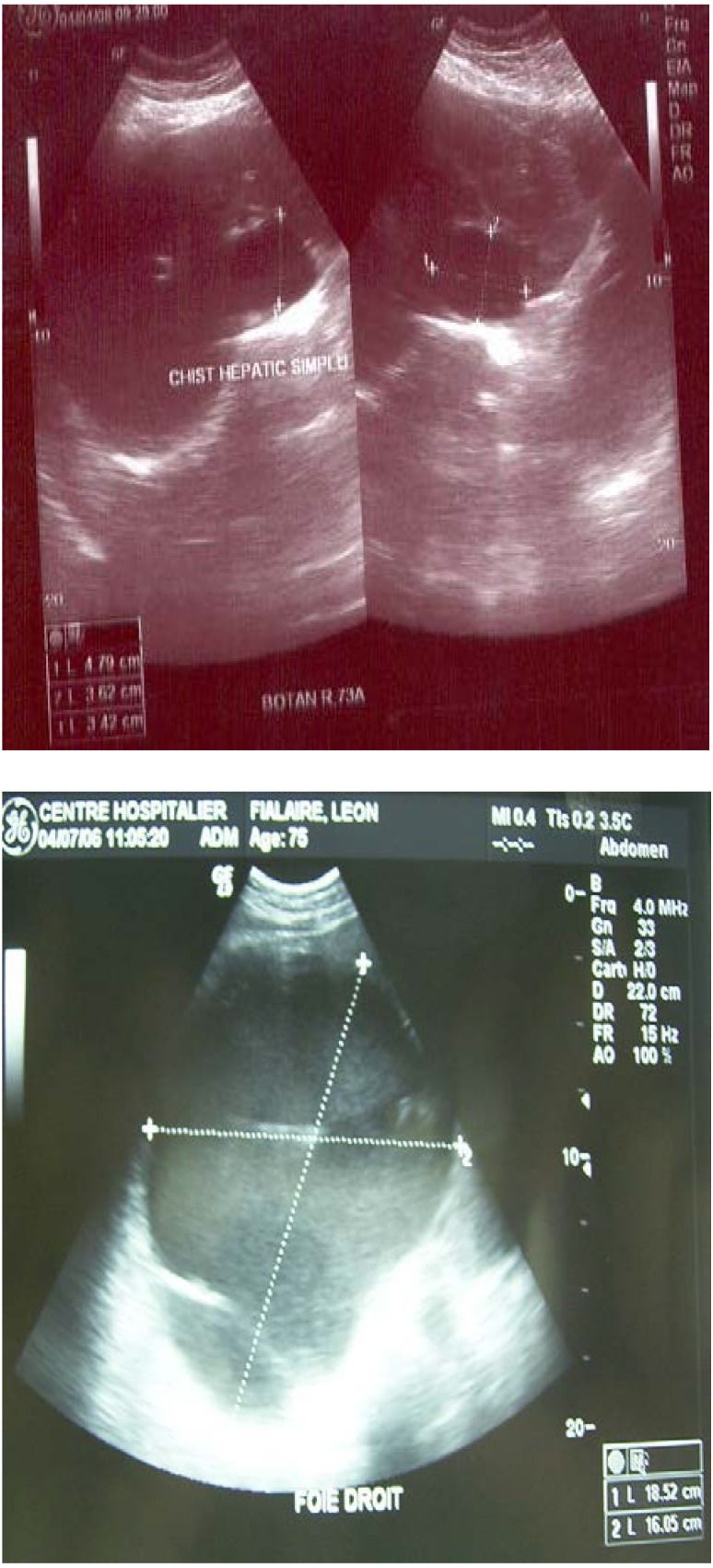
Ultrasound images from our study

Nuclear Magnetic Resonance has made significant contributions in the positive diagnosis of cystic liver disease, this investigation increased T2 signal identifying.

**Fig. 6, 7:**
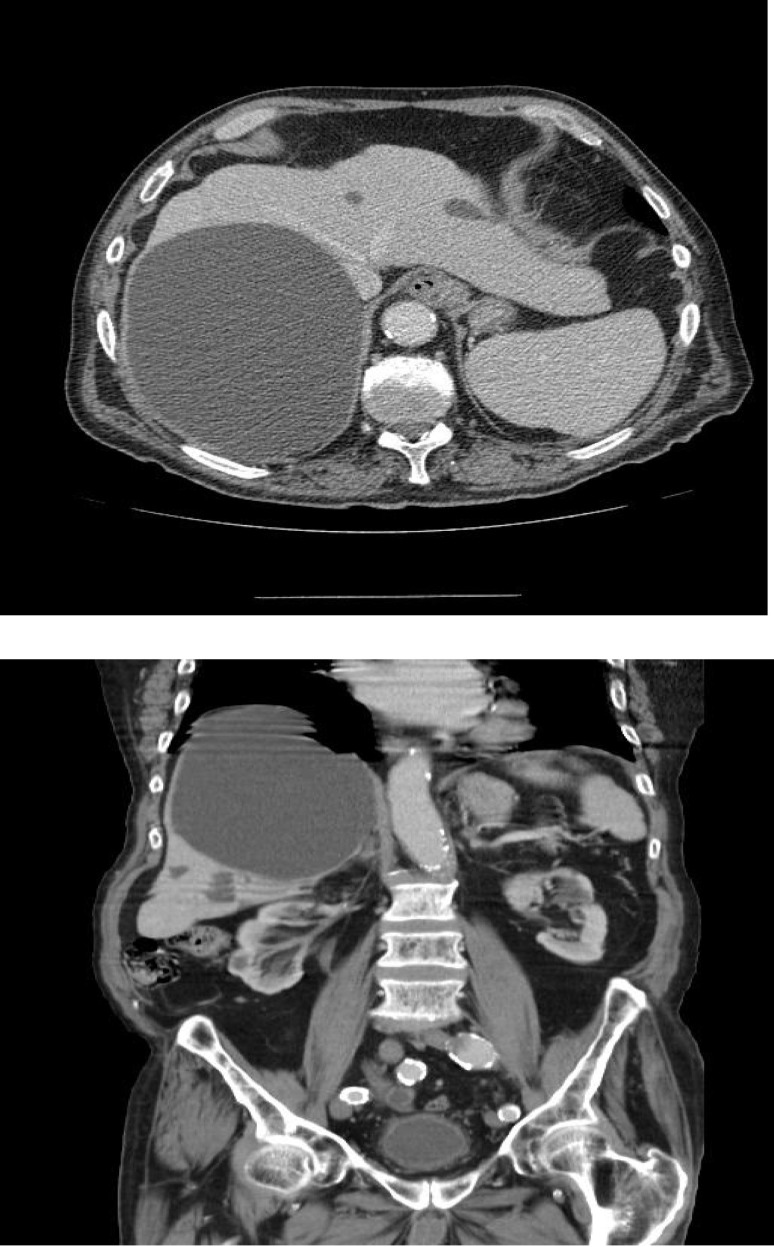
Images of CT in our research - simple cyst in the right lobe of the liver

Computed tomography proved less sensitive than liver ultrasound and nuclear magnetic resonance than showing round or oval fluid images without internal septa (**Fig. 6, 7**).

The average size of the cysts imaging in patients from our research was of 10cm, with values between 6 and 16cm.

Regarding the location of the cysts, most were located in the left lobe of the liver - 18 cases (75%), only 6 cases presented cystic lesions in the right hepatic lobe (modified).

**Fig. 8:**
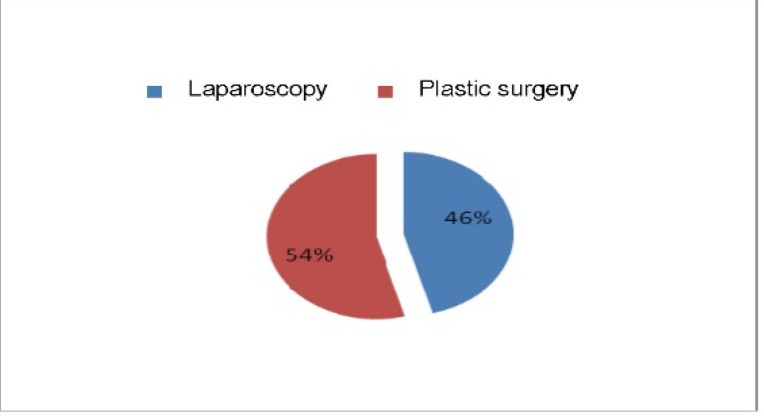
Types of therapeutic approach

Surgery is the wide fenestration (laparoscopy or laparotomy performed) and the remaining cavity drainage. In our study, 11 cases were operated by laparoscopic surgery and 13 by therapeutic approach (**Fig. 8**). 

Among patients who initially underwent laparoscopic approach (12 cases), in one case, it was necessary to convert to laparotomy.

The medium period for post operative hospitalization was specific to the patients who underwent a classical surgery - 8,5 days, and, for the ones who underwent a minimally invasive surgery, it was of 4,5 days. 

## Discussion

Liver cysts (and kidney) are controlled by chromosome 16, are more common in women than in men, have a right hepatic lobe predominant (83%) and become symptomatic after 60 years old. Only about 5-10% of the cysts cause symptoms [**3**].

In most cases, simple hepatic cysts are asymptomatic and discovered incidentally. Positive diagnosis is difficult especially for small cysts, which can be mistaken for hydatid cyst and biliary cystadenoma, whose surgery is different [**4**,**5**].

Literature attests that liver cysts are present predominantly in the right lobe of 83% [**6**]. The results of our research are in disagreement with literature; in our research, 75% of the treated cysts were located in the left hepatic lobe [**7**].

Literature attests more frequent in women than in men 2/ 1 (asymptomatic cysts) and 9/ 1 (symptomatic cysts) [**8**]. Our research findings were in agreement with literature data, 80% of the patients in our research being female.

About 50% of the cases were reported to be unique. Multiple cysts were sometimes associated with polycystic kidney disease [**9**]. 

Most cysts are asymptomatic. Even when they are large, they do not require surgery. In some cases, the main symptom meets the right upper quadrant pain caused by rapidly increasing the volume of bleeding cyst or intracyst [**10**]. In the research carried out by us, over 80% of the cases were asymptomatic for up to 10% of subjects introduced in the present study bleeding complications.

The surgical treatment for all the patients was represented by a large fenestration performed by laparotomy or laparoscopy accompanied by drainage cavity outstanding. Percutaneous aspiration was not recommended and was followed by rapid cyst recurrence. Puncture aspiration followed by administration of pure alcohol intracyst may be an alternative especially for patients who do not tolerate general anesthesia [**11**,**12**].

## Conclusions

• Laparoscopic fenestration of simple hepatic cysts is a simple, reproducible and efficient method with minimal surgical trauma and should represent the standard therapeutic approach for cystic liver disease.

• The limits of minimally invasive approach in hepatic cystic disease are the posterior localizations; the laparoscopic approach is easy for anterolateral location.

• The approach for simple liver cysts through laparotomy must remain a backup, this attitude followed by a relatively long period of hospitalization and a reduced quality of life compared with the minimally invasive approach.
